# Avulsion fracture of the posterior calcaneal tuberosity: anatomy, injury patterns, and an approach to management

**DOI:** 10.1007/s10140-025-02402-w

**Published:** 2025-10-15

**Authors:** Eric A. White, Alexander J. White, Matthew R. Skalski, MeNore G. Lake, Michael K. Chiu, Dani Sarohia, Nicholas A. Lewis, Dakshesh B. Patel

**Affiliations:** 1 Keck School of Medicine, University of Southern California, Los Angeles, CA USA; 2 Fusion Academy, Irvine, CA USA; 3 Skalski Chiropractic Radiology, Chippewa Falls, WI USA; 4 Los Angeles General Medical Center, Los Angeles, CA USA

**Keywords:** Calcaneal fracture, Avulsion, Trauma, Computed tomography, Radiography, Musculoskeletal

## Abstract

Calcaneal tuberosity avulsion fractures are often treated differently depending on several factors, including imaging diagnosis and classification. Timely identification of imaging findings, accurate interpretation, and effective communication can help avert serious clinical complications, including the necessity for soft tissue coverage and amputation. This article reviews the anatomy of the calcaneus, as well as the clinical and imaging findings of calcaneal tuberosity fractures. Imaging interpretation and clinical management of these fractures are discussed.

**Graphical abstract:**

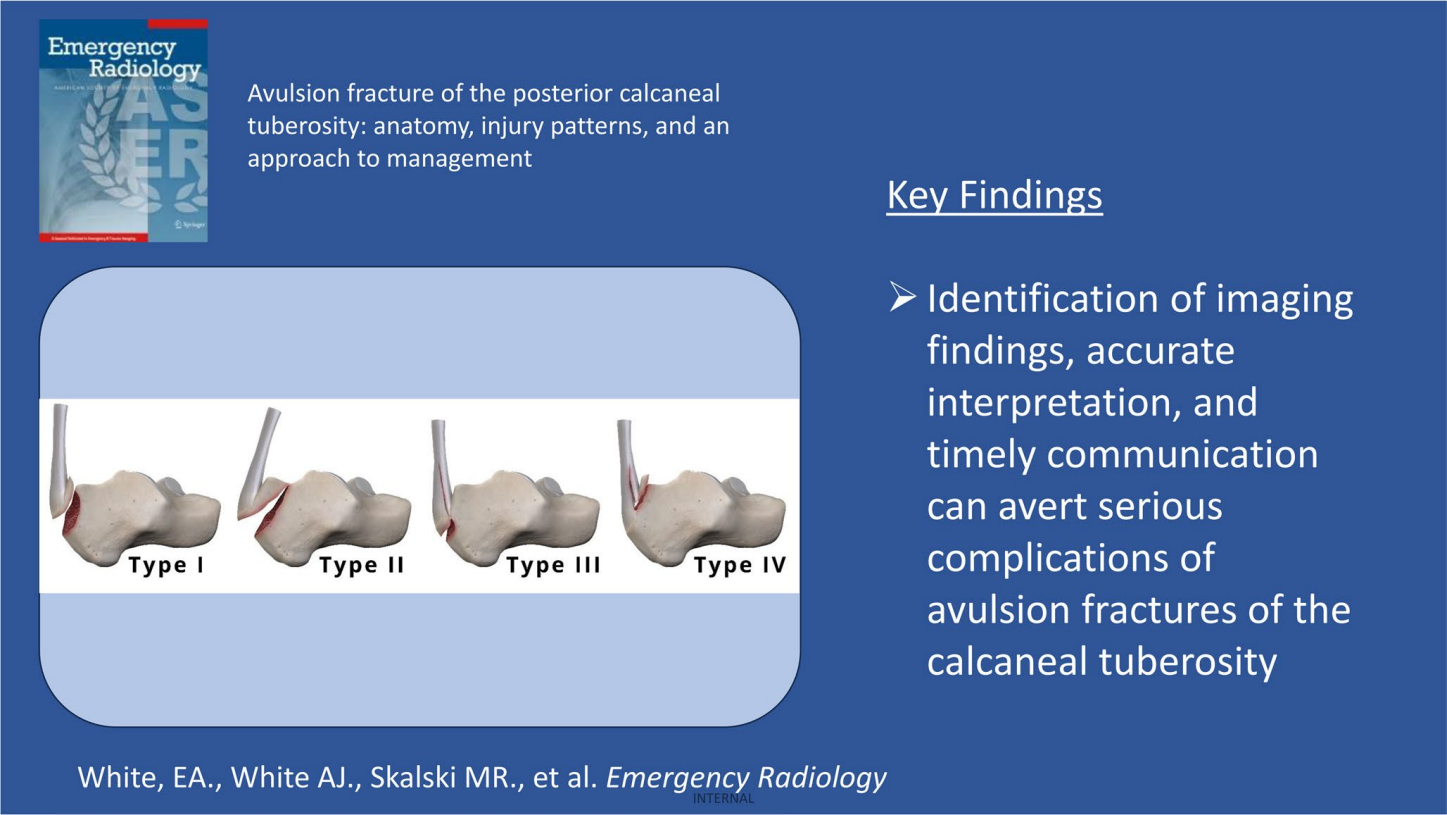

## Introduction

Avulsion fractures of the calcaneal tuberosity are considered relatively rare, constituting 1–3% of all calcaneal fractures [1, 2]. These injuries are often associated with risk factors such as increasing age, diabetes mellitus, and osteoporosis which can weaken bone structure and diminish resistance to tensile forces [3, 4]. The typical mechanism of injury involves forced dorsiflexion of a foot that is maximally plantar flexed [5] resembling the injury mechanism seen in Achilles tendon ruptures [6]. While both injuries share a similar etiology, to our knowledge a simultaneous tuberosity avulsion and complete Achilles tendon rupture has not yet been reported in the literature. Understanding posterior calcaneal tuberosity avulsion fractures is crucial for emergency radiologists because these injuries can have significant consequences if not promptly identified and communicated. Emergency radiologists are often the first to evaluate patients with these injuries, and their ability to recognize subtle findings, such as displaced fractures or soft tissue abnormalities, directly impacts patient outcomes. Timely and accurate diagnosis is essential to prevent complications like skin necrosis, malunion, or chronic functional impairment, which can arise from delayed treatment [4]. This article aims to provide a comprehensive review of the anatomy, injury patterns, and management strategies for posterior calcaneal tuberosity avulsion fractures, emphasizing the importance of early diagnosis and tailored treatment approaches.

### Anatomy and function

The calcaneal tuberosity is primarily made up of cancellous bone encased in a relatively thin cortical layer. The cancellous portion is made up of a complex network of trabeculae that is an important component for the overall strength of the bone [7]. The calcaneus acts like a lever for the gastrocnemius-soleus muscle complex, with the talus acting as the fulcrum during plantar flexion [8].

**Fig. 1:**
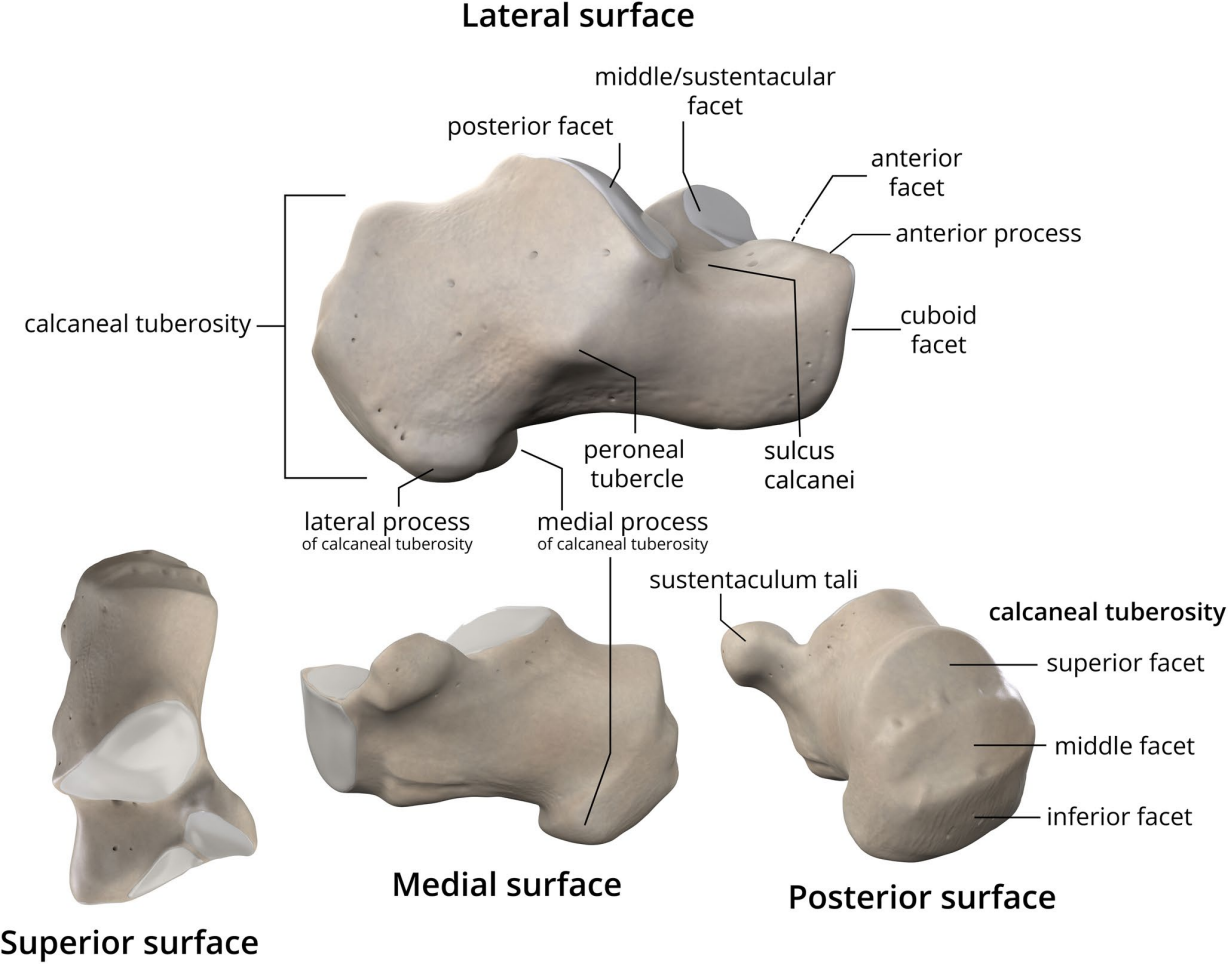
Normal anatomy of the calcaneus and calcaneal tuberosity

The Achilles tendon plays a critical role in transmitting forces generated by the gastrocnemius-soleus complex. It has a broad insertion on the middle third of the posterior calcaneal tuberosity (Fig. 1). The tendon has a broad insertion, measuring 1.2 to 2.5 cm in width. It has a unique coiled fiber pattern that rotates approximately 90 degrees before attachment [9, 10]. The structure provides viscoelastic properties, which enable the tendon to withstand forces that reach up to 12.5 times body weight during activities including running and jumping [11–13]. In cadaveric studies, differences of the Achilles tendon insertion have been shown to affect the fracture patterns observed in avulsion injuries [14, 15].

### Pathophysiology and mechanism of injury

**Fig. 2:**
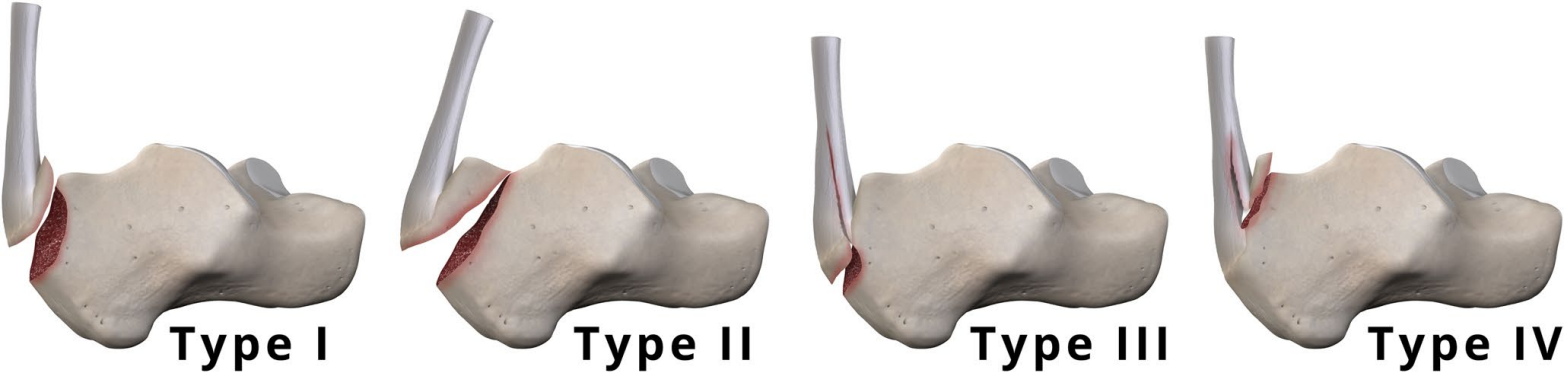
Calcaneal tuberosity avulsion fracture types (adapted from Lee et al. See text for details

Calcaneal fractures are typically classified into intra-articular and extra-articular types. Posterior calcaneal avulsion fractures are usually categorized as extra-articular [16, 17] without extending into the subtalar joint (Fig. 2). The strength of the bone is key in the development of avulsion fractures. Age-related declines in bone mineral density and strength, especially in postmenopausal women, can lead to a higher incidence of these injuries among women in the seventh decade of life [16, 18, 19].

The primary mechanism of injury typically involves a violent pull from the gastrocnemius-soleus muscle group, often with forced dorsiflexion. This can occur during low-energy events, such as tripping or pushing off from a standing position, or high-energy trauma, such as falling from a height [16, 20]. Hyperextension of the ankle or pushing off from a dorsiflexed foot may also result in this injury [21]. Blunt trauma resulting in this fracture has been reported [22]. An open calcaneal tuberosity fracture caused by a meat cleaver has also been reported [23].

Avulsion fractures may occur without a documented traumatic history in patients with diabetes mellitus and peripheral neuropathy. These patients may not feel these injuries due to reduced pain sensitivity, which can lead to delayed diagnosis and treatment [19, 24].

### Clinical presentation

**Fig. 3:**
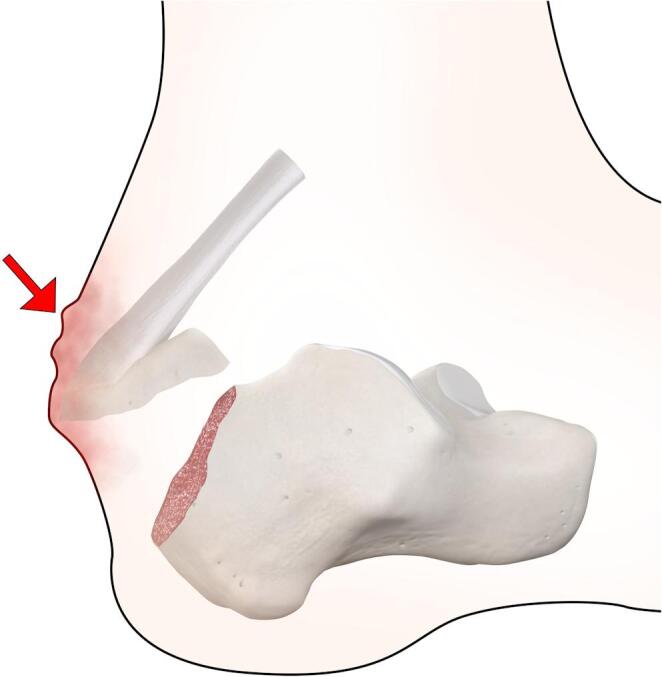
Graphic representation of soft tissue impingement. The force of the Achilles tendon pulls the calcaneal fragment proximally. This can result in impingement of the skin and soft tissues posteriorly (red arrow). Soft tissue findings include blister formation or skin breakdown

The typical presentation of a patient with a calcaneal tuberosity avulsion fracture is that of severe pain localized to the posterior heel. These patients often cannot bear weight on the affected foot. A common feature of these injuries is soft tissue compromise, where the displaced bony fragment can put significant pressure on the overlying skin. This can result in blanching, skin tension, and skin necrosis if untreated (Fig. 3). Elderly or diabetic patients may be at particular risk, as they may have poor vascular supply and delayed healing, which can worsen complications. Swelling and bruising around the posterior heel may be present, which can indicate the injury’s severity.

### Physical examination

On physical examination, the skin overlying the posterior calcaneus may appear pale or non-blanching, indicating increased pressure from the displaced fragment. This can lead to skin necrosis as well as the formation of skin ulcers if not rapidly addressed [19]. Functional tests may show an inability to fully plantarflex the foot [24]. The Thompson test (squeezing the calf muscle and observing for absent plantar flexion of the foot) may be positive. These findings suggest disruption of the Achilles tendon or its insertion [25]. Palpation may elicit tenderness over the posterior calcaneus, and the displaced fragment may be palpable in severe cases.

### Imaging and diagnosis

Imaging and diagnosis are critical for identifying posterior calcaneal tuberosity avulsion fractures and can determine the appropriate management. These fractures are often subtle and may not be detected or may be misdiagnosed. This may be seen especially in low-energy trauma cases or in patients with conditions like diabetes or osteoporosis. A detailed imaging approach is important to prevent treatment delays and complications such as malunion and skin necrosis.

### Radiographs and computed tomography

Radiographs are typically the first imaging study performed for suspected calcaneal fractures. The views obtained typically include lateral and axial (Harris) views of the hindfoot/calcaneus, as well as anteroposterior and oblique views of the foot. The lateral view is effective for identifying calcaneal tuberosity avulsion fractures [26–28]. This view will commonly demonstrate a fracture line that extends posteroinferiorly from the superior aspect of the calcaneal tuberosity to the posterior calcaneal surface, inferior to the Achilles tendon insertion.

Computed tomography (CT) provides improved visualization of the subtalar joint and can afford a detailed assessment of fracture patterns [29–31]. CT with multiplanar reformat enables accurate evaluation of fragment size and degree of comminution [32]. CT provides more accurate information regarding fracture size and displacement which may be underestimated or overestimated on radiographs. CT also offers improved visualization of surrounding soft tissues compared to radiography. Tendon entrapment, dislocation, or tearing may be seen, and has been identified in 16–25% of patients evaluated with CT for hindfoot fractures, but calcaneal avulsion fractures are not associated with these except for rare concurrent Achilles injury (mentioned in 2 case reports) [33–37] CT may also show fracture blisters that can be seen with calcaneal fractures [38].

### Magnetic Resonance Imaging (MRI)

While not routinely used for diagnosing calcaneal fractures, MRI can be invaluable in specific cases. It is very effective for assessing soft tissue injuries, including tears of the Achilles tendon [39]. In addition, MRI can reveal bone marrow edema and trabecular bone injuries indicating an occult fracture when radiographs and CT scans appear normal [40]. It can also identify conditions like tendinopathy or stress fractures, that may predispose patients to avulsion injuries [41].

### Other imaging modalities

Ultrasound may be employed to evaluate the integrity of the Achilles tendon after the radiographic detection of a calcaneal tuberosity fracture. Ultrasound is both sensitive and specific for assessing the Achilles tendon for partial or complete tears [42]. It can also show soft tissue swelling or hematoma formation, and ultrasound-guided needle aspiration may be beneficial in cases of suspected infection or hematoma.

Emerging imaging techniques, such as dual-energy CT (DECT) and quantitative MRI, are being explored for their potential to provide additional diagnostic information. DECT can differentiate between bone and soft tissue structures with greater accuracy [43]. DECT can identify bone marrow edema in the setting of trauma which may help identify subtle fractures [44] DECT had a good diagnostic performance for identifying Achilles tendon tears when compared with MRI [45]. Quantitative MRI can assess bone quality and tendon integrity [46, 47]. These modalities may play a role in the future management of calcaneal fractures, particularly in complex or recurrent cases.

### Classification of fractures

#### Lee classification

Lee et al. classified calcaneal avulsion fractures into four types, modifying the original classification proposed by Beavis et al. [17, 22]. These fractures as a group are illustrated in Fig. 2.

**Fig. 4:**
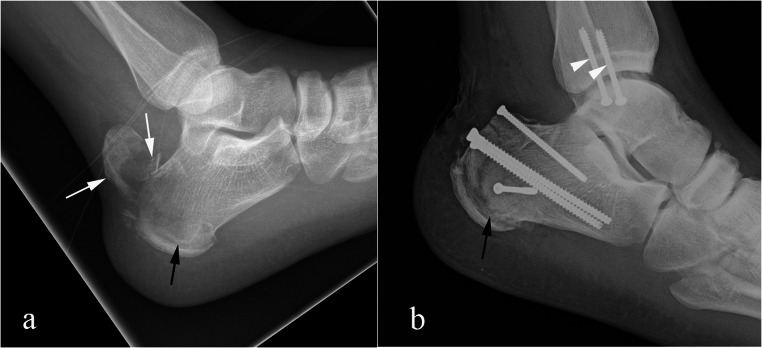
Type I calcaneal tuberosity avulsion fracture. 26-year-old man with a comminuted calcaneal tuberosity avulsion fracture (arrows) after a fall from height (**a**). Follow up (**b**) after fixation with multiple screws. The fracture extended inferiorly involving the entirety of the calcaneal tuberosity (black arrows in a and b). CT was not obtained but likely would have resulted in improved visualization of the inferior extent of the fracture (black arrows in a and b). Note is made of screw fixation (arrowheads) of a medial malleolus fracture in b

**Fig. 5:**
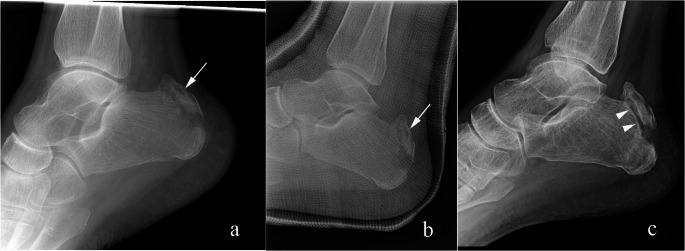
Type I calcaneal tuberosity avulsion fracture. 71-year-old woman with pain after a fall. Lateral radiographs show a calcaneal tuberosity fracture (arrows in a and b) at presentation (a) and at 6 weeks follow up (b). Due to comorbidities the patient was treated conservatively. At 3 months follow up (c), there is non-union of the fracture fragment (arrowheads) with no osseous bridging and sclerotic margins

**Fig. 6:**
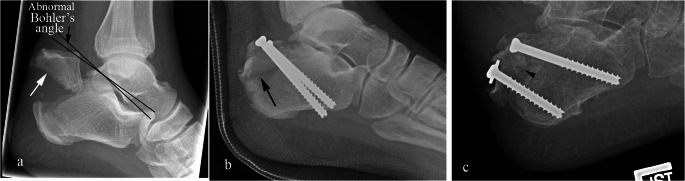
Type II calcaneal tuberosity avulsion fracture. Lateral radiographs of a 47-year-old man with calcaneal tuberosity avulsion fracture (white arrow) with an abnormal Bohler’s angle (black arrow) at presentation (**a**), clinically with skin tenting and blanching (not shown). Follow up radiograph (**b**) after percutaneous screw fixation resulted in persistent displacement of the fracture (black arrow in b). Follow up 11 months later (**c**) after revision with placement of new screws showing improved alignment of the osseous fragments (arrowhead)

**Fig. 7:**
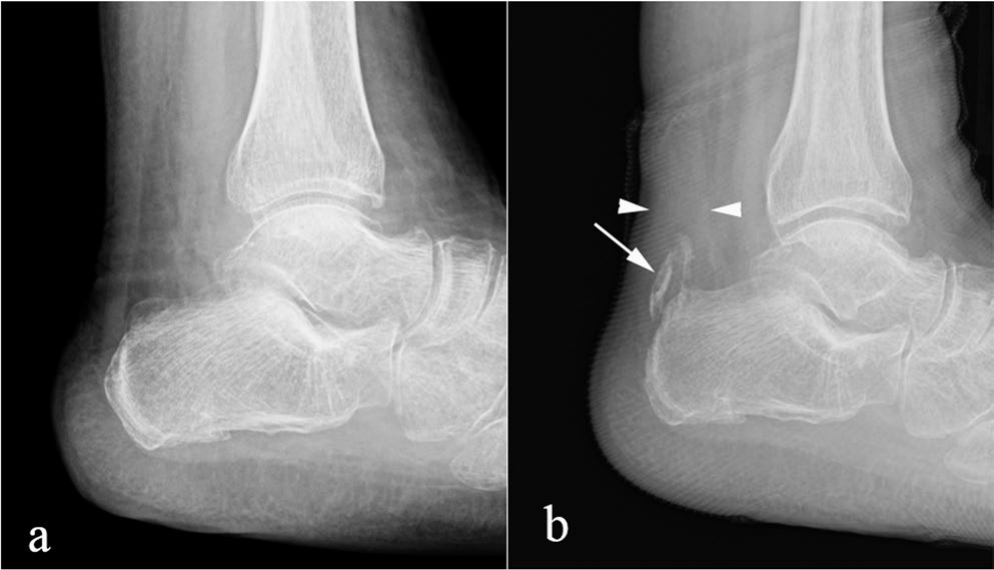
Type III calcaneal tuberosity fracture. 53-year-old woman initially presented after a slip and fall with a distal fibula fracture (not shown). Lateral radiograph (**a**) shows normal calcaneal tuberosity. Seven weeks later (**b**), the patient fell and complained of 8/10 heel pain. Lateral radiograph shows a calcaneal avulsion fracture (arrow in b) and thickening of calcaneal tendon shadow (between arrowheads in b). No further follow up images were obtained

**Fig. 8:**
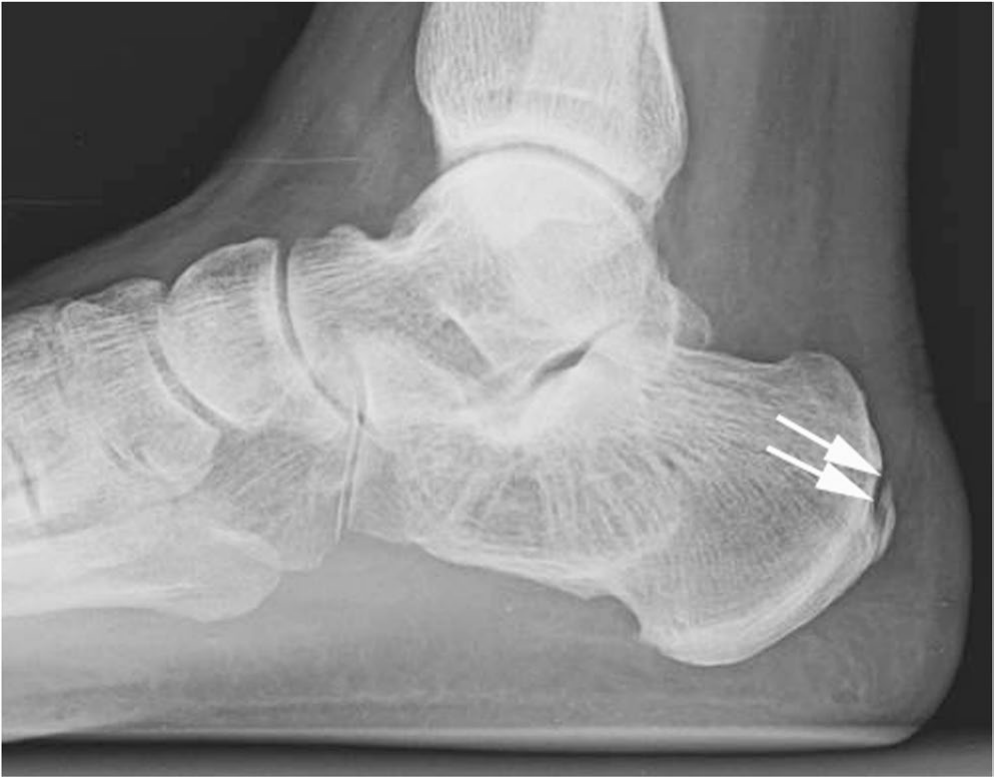
Type III calcaneal tuberosity fracture. 44-year-old man with pain after a fall. Lateral radiograph shows a mildly displaced avulsion fracture of the calcaneal tuberosity (arrow). The patient was treated conservatively, and no further imaging was obtained

- Type I: Simple extra-articular avulsion fracture (Figs. 4 and 5).

- Type II: “Beak” fracture characterized by an oblique fracture line extending posteriorly from just behind Bohler’s angle (Fig. 6).

- Type III: Infrabursal avulsed fracture from superficial fibers in the middle third of the posterior tuberosity (Figs. 7 and 8).

- Type IV: “Beak” fracture with a triangular fracture fragment separated by deep fibers only from the upper border of the tuberosity. This article does not include an example of a type IV fracture, which is considered rare [22].

All these fractures occur in osteopenic or osteoporotic bone. Beavis et al. concluded that the Type I “sleeve” fracture typically occurs in older patients with significant osteoporosis [17]. The fracture patterns are believed to arise from osteoporosis, the mechanism of injury, and fibers of the Achilles tendon that transmit the force [22].

#### de Soto classification

The Carnero-Martín de Soto classification categorizes calcaneal tuberosity avulsion fractures into two types based on the degree of fragment displacement.

- Type I: Fracture fragments are displaced by less than 2 cm. These fractures are associated with a 30% risk of complications following treatment.
- Type II: Fracture fragments are displaced by 2 cm or more. These injuries carry a significantly higher complication rate of 90%, with soft tissue complications being the most frequent [48].

Liu et al. suggest different imaging to minimize diagnostic oversight in calcaneal tuberosity avulsion fractures. Type II fractures, characterized by clear bony avulsion, are adequately assessed with X-ray and CT, which provide high-resolution visualization of fragment displacement. For Type I, III, and IV fractures, where bony defects are minimal or absent, MRI can provides additional information regarding Achilles tendon integrity, detection of soft tissue avulsion, and evaluation of the calcaneal tuberous cortex, which may demonstrate injury (edema or microfracture) apparent only on MRI [49].

### Differential diagnosis

**Fig. 9:**
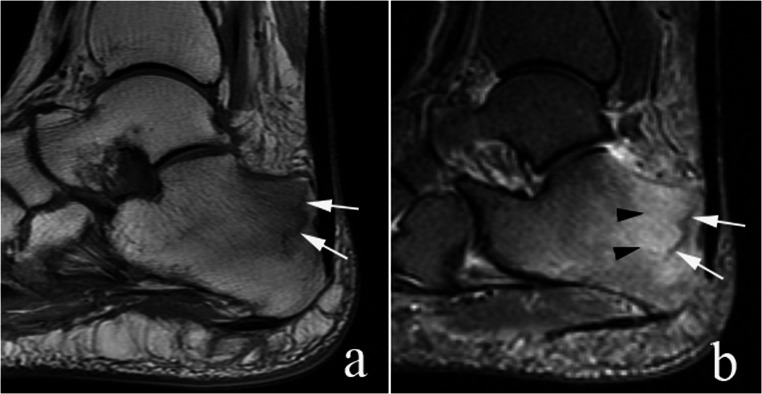
Stress reaction/stress fracture. 55-year-old woman with diabetes and heel pain. Sagittal T1 (**a**) and STIR (**b**) images show a calcaneal stress fracture (arrows in a. and b.) with adjacent edema-like signal (black arrowheads in b). The patient was treated conservatively and her pain resolved. No additional imaging was performed

The differential diagnosis would include stress reaction/stress fracture (Fig. 9) of the posterior aspect of the calcaneus, tennis leg, and Achilles tendon rupture. Stress reaction/stress fracture can be differentiated from calcaneal tuberosity avulsion fracture by the mechanism and clinical presentation as well as by imaging findings. Calcaneal stress reaction develops gradually due to repetitive microtrauma, commonly in athletes or in those with increased activity. The patient history does not include acute trauma [50]. The radiographic findings of stress reaction/stress fracture may be negative or may show linear sclerosis without a displaced fracture. MRI is more sensitive, showing bone marrow edema like signal with possible micro trabecular fracture, however no displaced fragment is present [50].

Tennis leg (injury injury of the myotendinous junction of the gastrocnemius-soleus muscle complex) could also present in a similar fashion. This typically presents clinically with acute mid-calf pain, a snapping sensation, swelling, ecchymosis, and tenderness, often at the myotendinous junction of the medial gastrocnemius head. Ultrasound and MRI typically show a fluid collection, usually tracking along the fascia and located between the medial gastrocnemius and soleus muscles [51].

Achilles rupture is diagnosed clinically with a patient history of a distinct “pop” or “giving way” sensation, and a positive Thompson test (calf compression fails to cause plantar flexion) [52]. Ultrasound and MRI show an Achilles tendinous gap with fluid in the defect, with possible retraction of the tendon ends [53].

### Approach to management

Non-surgical treatment is reserved for patients with minimal displacement or no displacement [19]. However, this approach can lead to complications such as malunion of the calcaneal tuberosity which may result in issues like skin irritation with footwear, reduced dorsiflexion, and challenges with activities like stair climbing [19]. Fractures without signs of skin tenting or blanching can be temporarily managed with splinting in plantarflexion and scheduled for non-urgent surgical fixation.

For fractures with significant displacement, surgical intervention is commonly performed within 2 weeks of injury [54]. In cases where the displaced fracture leads to skin tenting, blanching, or other signs of vascular compromise, urgent or emergent intervention is necessary to alleviate skin tension and prevent further complications, such as necrosis [55, 56]. During clinical evaluation, the posterior skin overlying the calcaneus should be carefully examined for signs of skin tenting and blanching. Blanching indicates increased pressure and reduced vascular supply, which can progress to partial or full thickness skin breakdown. When blanching or skin breakdown is present, emergent treatment is required (Figs. 5 and 6), and the orthopedic surgery service should be contacted immediately [56]. Treatment options for displaced fractures include percutaneous screw fixation or open reduction and internal fixation (ORIF) using a lateral approach.

The type of treatment can be assisted by the fragment size [57], as well as the classification described by Lee et al. [22]. Types I and II are larger fracture fragments, and are generally treated with screw fixation, while type III fractures include smaller fragments and are usually treated with suture anchor fixation and type IV fractures are treated conservatively [22]. Type I fractures typically occur in osteoporotic patients as the result of an insufficiency fracture. Cancellous screws should be used in this situation, to improve fixation in the osteoporotic bone. Type II fractures typically require emergency reduction to avoid skin necrosis [22]. Type III fractures involve only the superficial fibers of the Achilles tendon. For this reason, suture anchor fixation can be helpful to fix the small avulsed fragment. Type IV fractures involve only the deep fibers of the Achilles tendon and are typically minimally displaced. These can typically be managed conservatively [22]. The use of plates and screws is generally performed once the condition of the surrounding soft tissues allows for surgical intervention [58].

**Fig. 10:**
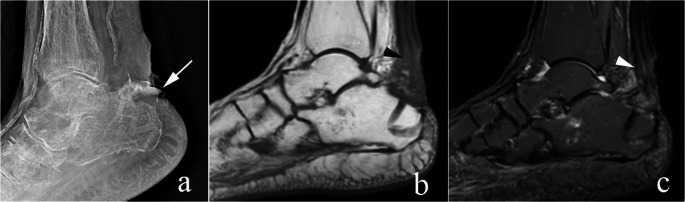
Complication - wound/skin break down. 62-year-old woman with diabetes, status post kidney transplant on prednisone and other immunosuppressants. The patient presented to an outside institution with an avulsion of the calcaneal tuberosity as seen on lateral radiograph (arrow in **a**.). This was debrided and repaired. Follow up MRI, with sagittal T1 (**b**) and short tau inversion (STIR, **c**) sequences show a thickened appearance of the Achilles tendon (arrowheads in b and c) after Arthrex BioComposite Achilles SpeedBridge repair. However, adequate skin coverage of the wound could not be obtained, and the patient subsequently developed infections, dehiscence, and wound breakdown. This ultimately resulted in below the knee amputation. No post amputation imaging was obtained

**Fig. 11:**
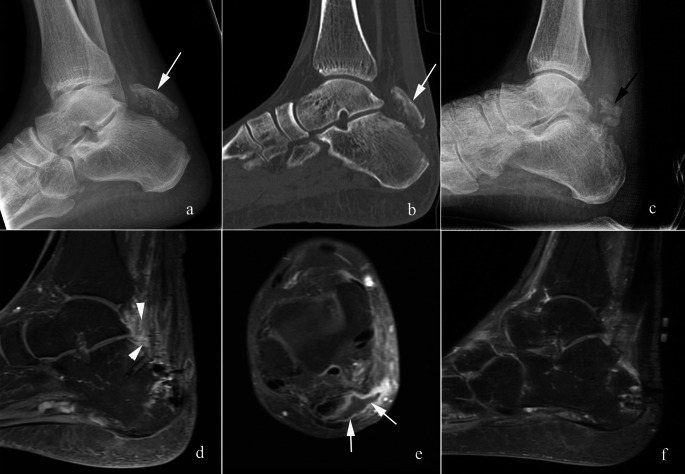
Complication – infection and failed Achilles tendon repair. 74-year-old man presented 2 weeks after an ankle injury demonstrating a displaced avulsion fracture (arrows in a and b) of the calcaneal tuberosity on lateral radiograph (**a**) and sagittal CT reformat (**b**). The patient went to surgery and underwent fragment excision with Achilles tendon repair with Arthrex BioComposite Achilles SpeedBridge. Two months later, post operative radiograph (**c**) shows osseous fragments in the surgical bed (black arrow in c). Post operative sagittal (**d**) and axial (**e**) T1 fat saturated post contrast images show phlegmonous changes (arrowheads in d) and a fluid collection adjacent to the Achilles tendon (arrows in e). The patient was treated with incision and drainage. Intraoperative diagnosis was failed Achilles tendon repair. The fiber tape and suture anchors were removed. Follow up sagittal STIR image (**f**) showed resolution of the fluid collection and no evidence of osteomyelitis. The patient is awaiting Achilles tendon reconstruction

Complications typically include skin necrosis, wound infection, osteomyelitis, malunion/nonunion (Fig. 5), and loss of Achilles tendon function [49, 59]. Examples of skin necrosis/breakdown, infection, and loss of Achilles tendon function are illustrated in Figs. 10 and 11.

## Conclusion

Avulsion fractures of the calcaneal tuberosity are injuries that can have devastating outcomes if not promptly and accurately managed. Radiologists play a critical role in detecting and characterizing these fractures, as accurate diagnosis can guide appropriate management and significantly improve patient outcomes. Early recognition and reporting of fracture features including fragment size, displacement, comminution, and associated tendon injuries are vital for guiding appropriate management. Additionally, radiologists should monitor follow-up imaging for fragment displacement or refracture to ensure proper healing and avoid further complications.

## Data Availability

Not applicable.
